# RNA-Seq Analysis in Non-Small Cell Lung Cancer: What Is the Best Sample from Clinical Practice?

**DOI:** 10.3390/jpm14080851

**Published:** 2024-08-11

**Authors:** Lorenzo Nibid, Giovanna Sabarese, Luca Andreotti, Benedetta Canalis, Daniela Righi, Filippo Longo, Margherita Grazi, Pierfilippo Crucitti, Giuseppe Perrone

**Affiliations:** 1 Research Unit of Anatomical Pathology, Department of Medicine and Surgery, Università Campus Bio-Medico di Roma, Via Alvaro del Portillo, 21, 00128 Roma, Italy; luca.andreotti@unicampus.it (L.A.); benedetta.canalis@policlinicocampus.it (B.C.); g.perrone@policlinicocampus.it (G.P.); 2 Anatomical Pathology Operative Research Unit, Fondazione Policlinico Universitario Campus Bio-Medico, Via Alvaro del Portillo, 200, 00128 Roma, Italy; g.sabarese@policlinicocampus.it (G.S.); d.righi@policlinicocampus.it (D.R.); 3 Research Unit of General Surgery, Department of Medicine and Surgery, Università Campus Bio-Medico di Roma, Via Alvaro del Portillo, 21, 00128 Roma, Italy; filippo.longo@policlinicocampus.it (F.L.); m.grazi@student.unisi.it (M.G.); p.crucitti@policlinicocampus.it (P.C.); 4 Thoracic Surgery Operative Research Unit, Fondazione Policlinico Universitario Campus Bio-Medico, Via Alvaro del Portillo, 200, 00128 Roma, Italy

**Keywords:** RNA-seq, NGS, precision medicine, molecular pathology, target therapy, pre-analytical variables, NSCLC

## Abstract

RNA-based next-generation sequencing (RNA-seq) represents the gold standard for detecting gene fusion in non-small cell lung cancer (NSCLC). Despite this, RNA instability makes the management of tissue samples extremely complex, resulting in a significant number of test failures with missing data or the need to switch to other techniques. In the present study, we analyzed pre-analytical variables in 140 tumor tissue samples from patients affected by NSCLC to detect features that increase the chances of successful RNA-seq. We found that the success rate of the analysis positively correlates with the RNA concentration and fragmentation index. Interestingly, small biopsies were more suitable samples than surgical specimens and cell blocks. Among surgical specimens, wedge resections demonstrated better results than lobectomy. Moreover, samples stored for less than 30 days (1 month) had a better chance of success than older samples. Defining the role of pre-analytical variables in RNA-seq allows the detection of more suitable samples for analysis and more effective planning of molecular-based diagnostic approaches in NSCLC.

## 1. Introduction

Molecular targeted therapies in solid tumors are currently based on the availability of molecular testing, which should be both time- and tissue-efficient. Target mutations and rearrangements can be detected using quantitative/real-time polymerase chain reaction (q-PCR), reverse-transcriptase polymerase chain reaction (RT-PCR), fluorescence in situ hybridization (FISH), DNA-based next-generation sequencing (DNA-seq), RNA-based next-generation sequencing (RNA-seq), and/or screened via immunohistochemistry, and each test can be performed on one or more platforms. As standardized criteria for molecular diagnostics are not available, the choice of test, platform, or sample type, together with the timing of the test, depends on the capabilities of the single center; therefore, the reproducibility and success of the analysis are not guaranteed [1,2].

With the advent of next-generation sequencing (NGS) techniques, it is possible to analyze a wide panel of genes, reducing costs and increasing the accuracy of the analysis. In 2020, the European Society of Medical Oncology (ESMO) published recommendations on the use of NGS for patients with advanced non-squamous non-small cell lung cancer (NSCLC), prostate adenocarcinoma, ovarian carcinoma, and cholangiocarcinoma [3]. Considering NSCLC, at the time of diagnosis, all advanced non-squamous NSCLC should be screened for EGFR (exon 18: G719x; exon 19: ex19del; exon 20: T790M, S768I, ex20ins; exon 21: L858R, L861Q), KRAS (G12C), BRAF (V600E), and HER2 mutations, for MET exon 14 skipping, and for ALK, ROS1, RET, NTRK1, NTRK2, and NTRK3 rearrangements [4].

Although DNA is a highly stable molecule, its study has some limitations, mainly due to mutations in non-coding DNA and epigenetics [1]. In addition, in DNA-seq, the detection of gene fusion can be complicated by the presence of large intronic regions, which are often difficult to amplify and sequence. Conversely, RNA-seq detects fusions that undergo transcription and analyzes mature mRNA that is not affected by intron size. RNA-seq can provide better results than DNA-based testing in the detection of point mutations, insertions, or deletions that alter gene splicing [5]. Therefore, although RNA-seq represents the gold standard for detecting gene fusion, the reliability of ESMO recommendations in real-life clinical practice is not straightforward, especially considering the significant number of molecular testing failures. The instability of RNA makes extraction extremely complex, and suitable samples for histopathological diagnosis often result in the inadequate quantification of transcripts. Therefore, even if the RNA-seq approach seems to overcome the capabilities of DNA-seq, it is less commonly applicable in clinical practice.

Currently, knowledge is lacking on the impact of individual variables that adversely affect tumor sample quality in RNA-seq. Knowing more about these factors can allow for a better selection of suitable samples for RNA-seq, especially in NSCLC, in which a great number of genes should be tested, more often from small tissue specimens.

In the present study, we investigated the feasibility of RNA analysis in patients affected by NSCLC, focusing on the most relevant pre-analytical variables that can affect the success of analysis on formalin-fixed and paraffin-embedded (FFPE) samples. In detail, we related the sample site, RNA concentration and fragmentation index, and the storage time and sample type to the RNA-seq success rate. We also searched for variables affecting the RNA fragmentation index and for selection criteria to detect optimal samples for RNA-seq.

## 2. Materials and Methods

Tissue samples from 140 patients affected by NSCLC and selected to undergo molecular profiling according to the current clinical standard were evaluated for molecular testing between December 2021 and January 2023 at the Pathology Unit of Campus Bio-Medico University Hospital Foundation of Rome. We tested surgical specimens, cell blocks, or small biopsies taken from the primary tumor (T), lymph node site (N), or metastatic site (M). Bone biopsies were decalcified using EDTA. No patients received neoadjuvant treatment before molecular testing.

### 2.1. RNA Extraction

RNA was isolated using the High Pure FFPE RNA isolation kit (Roche Diagnostics, Mannheim, Germany, REF 04823125001) according to the manufacturer’s protocol. Briefly, the first step was dewaxing 10 µm-thick sections of FFPE tissue in a reaction tube with xylene and absolute ethanol. The samples were digested in RNA tissue lysis buffer, 10% SDS, and proteinase K overnight at 55 °C with shaking at 600 rpm. The next day, the samples were processed with DNase at room temperature and, after washing, were finally eluted with a specific buffer provided with the kit. The concentration and fragmentation of total RNA were determined using the Myriapod NGS Cancer Panel RNA kit and q-PCR.

### 2.2. Molecular Analysis

We used the Myriapod NGS Cancer Panel RNA NG034 (48 test, CE-IVD) kit from Diatech Pharmacogenetics (Ancona, Italy) that allows the preparation of NGS libraries for Illumina platforms. The fragmentation and concentration were analyzed using q-PCR to assess the accuracy of the RNA evaluation. For RT-PCR analysis, EasyPGX ready ALK/ROS1/RET/MET exon skipping and EasyPGX ready NTRK1/NTRK2/NTRK3 (CE-IVD Kit, Diatech Pharmacogenetics) were adopted. The row data were further evaluated with EasyPGX Analysis Software 4.0.16.

### 2.3. Fragmentation Index Cut-Off

**Figure 1:**
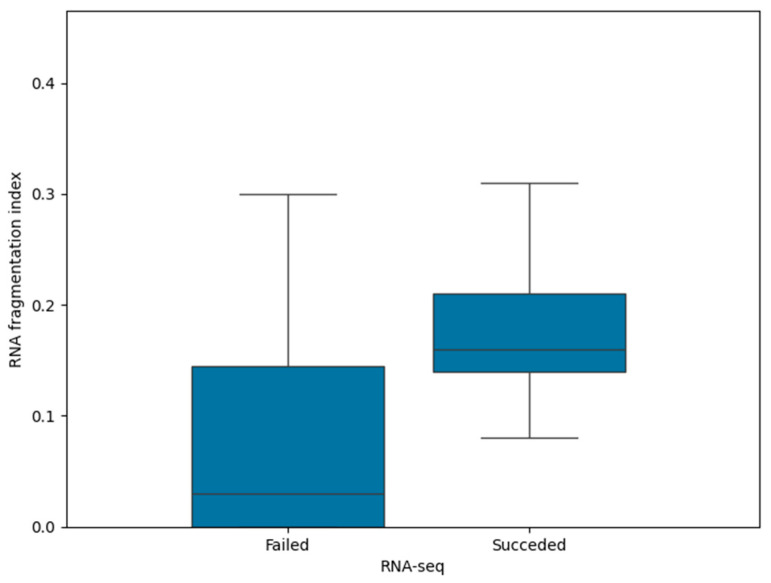
RNA fragmentation index in failed and successful samples tested using RNA-seq. The 75th percentile of failed samples corresponds to the 25th percentile of successful samples (0.14). Black bars within the boxes indicate the median value.

We focused on 102 samples tested using RNA-seq to detect an RNA fragmentation index cut-off and to discriminate between optimal and suboptimal samples. Therefore, we first evaluated the 25th and 75th percentiles of the RNA fragmentation index in successful and failed samples, which were 0.14 and 0.21 in the first group and 0.00 and 0.14 in the second group (Figure 1). As the 25th percentile of successful samples corresponds to the 75th percentile of failed samples, the cut-off value of 0.14 was found to discriminate between the optimal and suboptimal specimens to be tested using RNA-seq. The detected cut-off value was further applied to the 140 enrolled tissue samples to detect how pre-analytics affect RNA fragmentation.

### 2.4. Pre-Analytical Variables

Hematoxylin–eosin tissue slides of all enrolled samples were reviewed by two lung pathologists to assess the tissue availability for molecular testing, and pre-analytical variables were noted. In detail, we considered the type of specimen (i.e., surgical specimen, cell block, or small biopsy), the type of biopsy or surgery (endobronchial ultrasound [EBUS], esophageal endoscopic ultrasound [EUS], transthoracic computed tomography [CT], ultrasound-guided biopsy, lobectomy, wedge resection, thoracoscopic biopsy, lymphadenectomy, and metastasectomy [the surgical removal of metastases with adrenal and bladder localization]), the target of sampling (T, N, or M), the histotype (i.e., adenocarcinoma, squamous cell carcinoma, or other histotypes), the RNA concentration, and fragmentation values.

Moreover, we noted the fixation time and storage time. We referred to the fixation time as the elapsed time from the biopsy or surgery date to the sampling date in days. We obtained the fixation time using the formula:

$$Fixation\;time=\left(Sampling\;date\;-Biobpy/Surgery\;date\right)\;+\;1\;day\;*$$

where the sampling date is the date on which the histological specimen was sampled by a pathologist and transferred in a histology cassette to start the processing phase.

* The addition of 1 day in the formula is because each sample has fixation and processing times covering at least the span of 1 day.

We referred to storage time as the elapsed time from biopsy or surgery date to molecular analysis. We obtained the storage time using the formula:

$$Storage\;time=\left(NGS\;RNA\;analysis\;date-Biopsy/Surgery\;date\right)$$

### 2.5. Statistical Analysis

RNA concentration and fragmentation index, fixation time, and storage time were analyzed to verify the correlation with success rate (SR) through Spearman testing. We used Pearson’s chi-squared to evaluate the relationship between SR and sample type. The Kruskal–Wallis test was adopted to evaluate the difference between tumor sites or surgical/biopsy procedures in terms of the RNA concentration and fragmentation index. The significance was set at *p* < 0.05. Statistical analyses were conducted in SPSS software (IBM SPSS Statistics 27, Armonk, NY, USA).

## 3. Results

### 3.1. Sample Data

We recorded the pre-analytical variables of 140 FFPE tissue samples from consecutive patients affected by NSCLC who were evaluated to be tested via RNA-seq between December 2021 and January 2023. Among these, 114 (81.4%) were in-house samples, whereas 26 (18.6%) came from other institutions. Of the 140 samples, 102 (72.9%) were judged suitable for molecular testing according to the Myriapod NGS Cancer Panel RNA datasheet (low concentration index <0.063 ng/µL and low fragmentation index < 0.05), whereas 38 (27.1%) were primarily tested using RT-PCR based on the pre-test assessment of histopathological sample size. Molecular data were obtained in 54/102 (52.9%) patients tested using RNA-seq, whereas 27/102 (26.5%) specimens were further tested using RT-PCR. Two (7.4%) of the samples failed in RT-PCR, resulting in an SR of 92.6% (25/27). In 21/102 (20.6%) samples that failed RNA-seq, other driver mutations were found through DNA-seq or q-PCR, and no further analyses were performed to detect gene fusions. In samples judged inadequate for RNA-seq, RT-PCR succeeded in 28/38 (73.7%) and failed in 10/38 (26.3%) samples. Overall, in 12/140 (8.6%) samples, no molecular data on gene fusion were available because of a failure of the RNA-seq and/or RT-PCR.

Lung adenocarcinoma was the most frequent histotype evaluated for RNA-seq (124/140; 88.6%), followed by non-adeno/non-squamous histotypes (14/140; 10%) and squamous cell carcinoma of the lung (2/140; 1.4%). The group of non-adeno/non-squamous histotypes includes adenosquamous lung cancer, large cell lung cancer/NSCLC, Not-Otherwise Specified, pleomorphic carcinoma, and non-small cell neuroendocrine carcinoma of the lung.

Among the 140 samples, 102 (72.9%) were classified as primary NSCLC cancers and 38 (27.1%) as metastatic lesions from NSCLC. Among the metastatic lesions, 24 (63.2%) were distant metastases and 14 (36.8%) were lymph node metastases. Considering the sample type, 69 (49.3%) samples were biopsies, 63 (45%) were surgical specimens, and 8 (5.7%) were cell blocks. Moreover, we were able to obtain data on fixation time for 116 (83%) of the specimens. NSCLC samples were formalin-fixed for a median 2 days, but 21/56 (37.5%) surgical specimens and 41/60 (68.3%) cell blocks and small biopsies had a suboptimal fixation time (>2 days for surgical specimens and >1 day for small biopsies) [6,7]. Table 1 presents the baseline characteristics of the tissue samples.

### 3.2. RNA-Seq Data

A total of 102 (72.9%) samples were judged suitable for RNA-seq (Illumina MiSeq, San Diego, Canada, USA). The SR of the analysis was 52.9% (54/102). In 41/54 (75.9%) samples, no fusions were found, whereas ALK rearrangements were detected in 6/54 (11.1%) samples, MET exon skipping in 5/54 (9.3%) samples, ROS fusions in 1/54 (1.9%) samples, and RET fusions in 1/54 (1.9%) samples.

Among samples tested using RNA-seq, the SR was 61.1% (11/18) for tumors obtained from distant metastases, 53.4% (39/73) for tumors obtained from primary sites, and 36.4% (4/11) for tumors obtained from lymph node metastases. Regarding distant metastasis, the SR was 50% (2/4) for bone biopsies and 64.3% (9/14) for other sites. Considering sample type and sampling procedures, the SR was 60.3% (35/58) in small biopsies. The SR was 4.7% (11/17) in US-guided biopsies, 62.5% (10/16) in EBUS/EUS biopsies, 55.6% (5/9) in thoracoscopic biopsies, and 50% (6/12) in CT-guided biopsies. In surgical specimens, the SR was 48.7% (19/39). Interestingly, RNA-seq analysis performed better in samples from wedge resection than samples from lobectomy (10/15, 66.6% vs. 5/17, 29.4%). Regarding cell blocks obtained from fine needle aspiration, no data were obtained in the RNA-seq analysis (0/5; SR = 0%). Pearson’s chi-squared revealed significant differences in the SR among cell blocks, biopsies, and surgical specimens (*p* = 0.028).

We noted that the SR was higher with a higher RNA concentration and fragmentation index; therefore, the RNA concentration (*p* < 0.0001; r = 0.509) and fragmentation index (*p* < 0.0001; r = 0.556) positively correlate with RNA-seq success. In turn, RNA concentration appears to be related to the RNA fragmentation index (*p* < 0.0001; r = 0.445). RNA concentrations, isolated from each NSCLC sample, are reported in Table S1. Moreover, samples with a fragmentation index ≥ 0.14 had better results in terms of the SR. Molecular data were obtained in 42/55 (76.4%) RNA-seq tested tissue samples with a fragmentation index ≥ 0.14, compared with 12/47 (25.5%) samples with a fragmentation index < 0.14.

**Figure 2:**
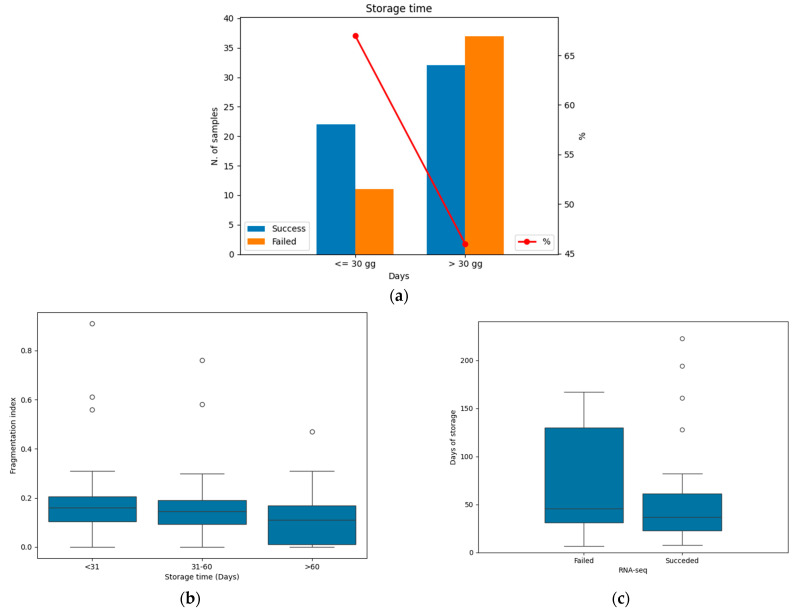
Relationship between storage time and RNA-seq success rate (SR). (**a**) Successful and failed RNA-seq tests before and after 30 days. The red line represents the SR in the two groups. Blue: successful samples; orange: failed samples. (**b**) Box plot representing the decrease in RNA fragmentation index over time. (**c**) Box plot representing the distribution of successful and failed RNA-seq tests depending on storage time. Black bars within the boxes indicate the median value.

Considering storage time, the SR decreases in samples tested after 1 month, with molecular data obtained in 22/33 (66.6%) samples tested within 30 days from the surgery/biopsy. The SR decreased to 32/69 (46.4%) in samples tested after 30 days (Figure 2a). Although no significant level was reached (*p* = 0.056) in terms of SR, a negative correlation was found between the storage time (samples tested before and after 1 month) and the RNA fragmentation index (*p* = 0.037; r = −0.206). The RNA fragmentation index decreased over time (Figure 2b). Figure 2c shows that, in most of the cases, failed samples were stored for longer than successful samples (median 46 vs. 37 days). Furthermore, the 25th and 75th percentiles of storage time were 31 and 130 days in failed samples and 23 and 61 days in successful samples, respectfully. All pre-analytical variables and relative SRs are shown in Table 2.

### 3.3. Estimation of Fragmentation Index

**Figure 3:**
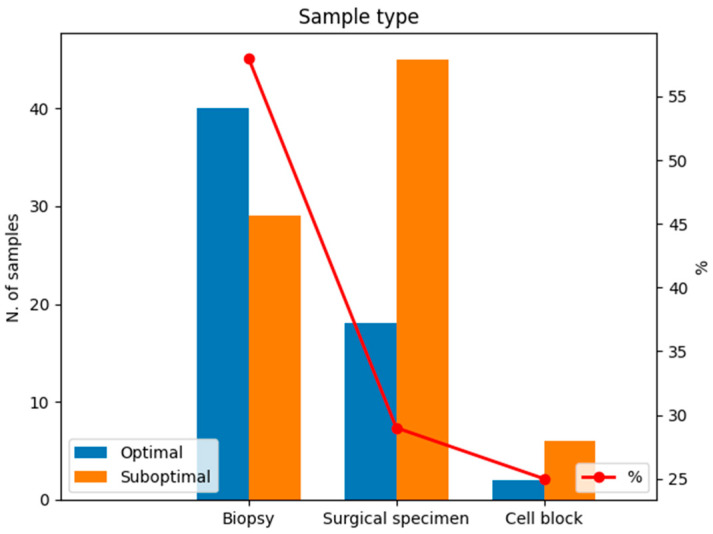
Optimal and suboptimal specimens in terms of the RNA fragmentation index considering sample type. The red line represents the percentage of optimal samples in terms of RNA fragmentation. Blue: optimal samples with RNA fragmentation index ≥ 0.14; orange: suboptimal samples with RNA fragmentation index < 0.14.

We evaluated the pre-analytical variables of the 140 tissue samples by adopting the cut-off value of 0.14 to define optimal and suboptimal samples for RNA-seq. Most tissue samples from biopsies were optimal in terms of the fragmentation index (40/69; 57%), whereas the minority of samples from surgical specimens or cell blocks had a fragmentation index ≥ 0.14 (18/63, 29% and 2/8, 25%, respectively; Figure 3). Thus, significant differences were found between the sample type and RNA fragmentation index (*p* = 0.002).

**Figure 4:**
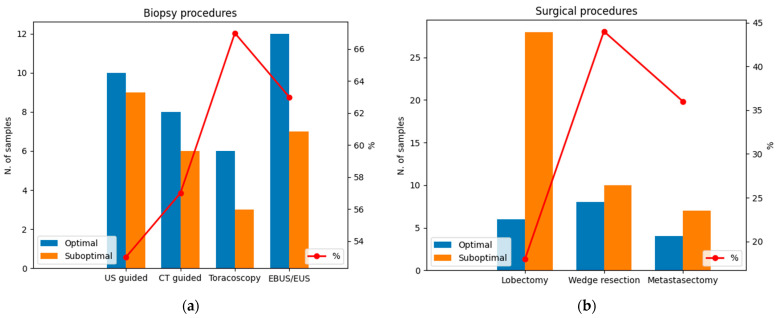
Optimal and suboptimal specimens in terms of the RNA fragmentation index considering (**a**) biopsy procedures and (**b**) surgical procedures. Note that, in biopsy procedures, the number of samples with optimal RNA fragmentation is higher than the number of suboptimal samples. Conversely, surgical procedures are related to a higher number of suboptimal specimens in terms of the RNA fragmentation index. The red line represents the percentage of optimal samples in terms of RNA fragmentation. Blue: optimal samples with RNA fragmentation index ≥ 0.14; orange: suboptimal samples with RNA fragmentation index < 0.14.

Considering biopsies, samples obtained from thoracoscopic biopsies are more likely to have an optimal fragmentation index (6/9, 67%), followed by endoscopic biopsies (EBUS/EUS; 12/19, 63%), CT-guided biopsies (8/14, 57%), and US-guided biopsies (10/19, 53%; Figure 4a). However, no significant differences were found between biopsy procedures and the RNA fragmentation index (*p* = 0.967). Moreover, samples from the thoracoscopic biopsies showed the best results in terms of RNA concentration (mean = 65.9 µg/mL), followed by US-guided and endoscopic (EBUS/EUS) biopsies (mean = 17.4 µg/mL and 17.2 µg/mL, respectively) and CT-guided biopsies (mean = 4.4 ng/mL). Therefore, significant differences were found between biopsy procedures and RNA concentration (*p* = 0.017). Interestingly, wedge resections were optimal samples in terms of the fragmentation index; values ≥ 0.14 were recorded in 8/18 (44%) samples from wedge resection, in 4/11 (36%) samples from metastasectomy, and 6/34 (18%) samples from lobectomy. Therefore, significant differences were found between surgical procedures in terms of the RNA fragmentation index (*p* = 0.028; Figure 4b).

**Figure 5:**
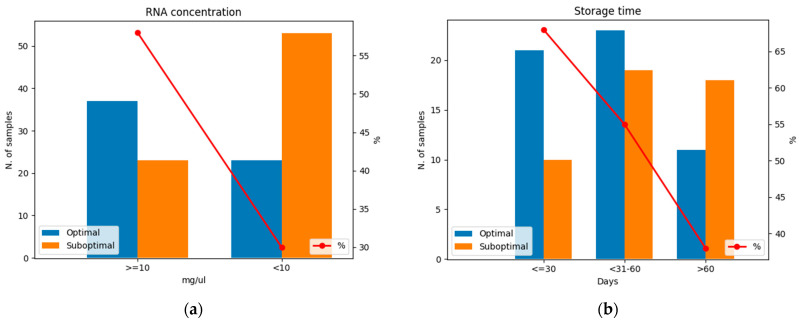
Optimal and suboptimal specimens in terms of the RNA fragmentation index considering (**a**) RNA concentration and (**b**) storage time. Note that, under the concentration index of 10 ng/µL and >60 days of storage, samples were more likely to have a suboptimal RNA fragmentation index. The red line represents the percentage of optimal samples in terms of RNA fragmentation. Blue: optimal samples with RNA fragmentation index ≥ 0.14; orange: suboptimal samples with RNA fragmentation index < 0.14.

Moreover, the RNA concentration and fragmentation index are directly related; an optimal fragmentation index was observed in 37/64 (58%) specimens with RNA concentrations ≥ 10 ng/µL and in 23/76 (30%) specimens with RNA concentrations < 10 ng/µL (Figure 5a). Considering the length of storage (storage time), we noted that RNA fragmentation decreases over time. In samples tested within 30 days from the diagnostic procedure, we registered 21/31 (68%) specimens with an optimal RNA fragmentation index, whereas in samples tested between 31 and 60 days and >60 days, an optimal RNA fragmentation index was found in 23/42 (55%) and 11/29 (38%) specimens, respectively (Figure 5b).

**Figure 6:**
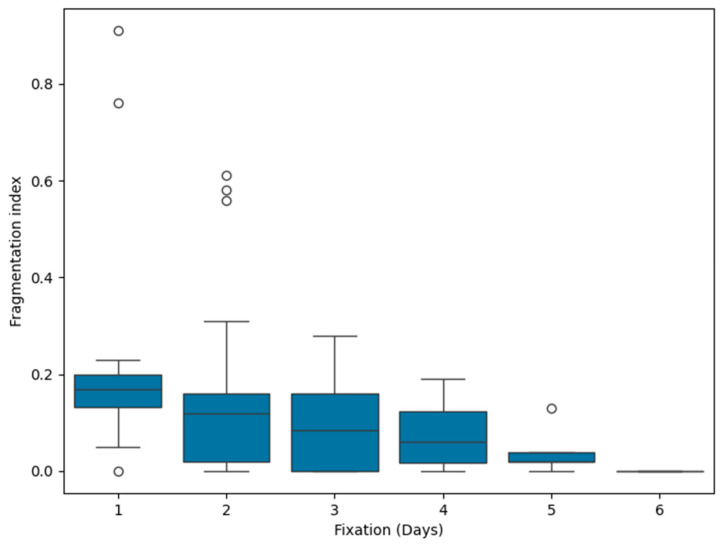
Changes in the RNA fragmentation index over time. Black bars within the boxes indicate the median value.

Moreover, we found that the RNA fragmentation index inversely correlates with fixation time (*p* < 0.001, r = −0.333). Figure 6 presents the reduction in the RNA fragmentation index over time.

No significant differences were found between the primary and metastatic samples (*p* = 0.992); the optimal values were recorded in 43/101 (42%) primary tumors, 10/24 (42%) distant metastases, and 6/14 (40%) lymph node metastases.

## 4. Discussion

The performance of molecular testing relies not only on the quality of the adopted technique, but also on the quality of the tested specimen, which is, in turn, determined by pre-analytical variables. Thus, according to the “garbage in, garbage out” principle, testing suboptimal samples implies that suboptimal results will be obtained [2,8].

Recognizing the widespread and growing importance of molecular data derived from patient specimens, the Personalized Healthcare Committee (PHC) of the College of American Pathologists (CAP) established the Pre-analytics for Precision Medicine Project Team (PPMPT) to develop a basic set of evidence-based recommendations for pre-analytics that can be implemented in routine pathology practice [9]. Among the pre-analytical variables affecting molecular testing, “warm” and “cold” ischemia time, tissue decalcification, storage conditions (i.e., humidity, temperature), length of storage, specimen size, and fixation time are reported in the literature [9,10,11]. As these variables have been explored mostly in the context of RT-PCR, q-PCR, or DNA-based NGS, their role in RNA-seq remains unclear [11,12].

In the present study, we investigated RNA fragmentation and the concentration index of 140 NSCLC tissue samples to establish the optimal conditions for NGS analysis. In addition, we investigated pre-analytical variables that affect RNA-seq results in real-life clinical practice. We tested 102 FFPE samples using RNA-seq, obtaining an SR of 52.9%. No differences were noted between the primary and secondary sites in terms of SR and the RNA fragmentation index (*p* = 0.992).

Previous studies demonstrated that DNA-based NGS analysis performs better with surgical specimens than small biopsies. A SR of 97% in 614 tissue samples obtained via resection using a 50-gene hotspot mutation panel was described [11]. Similarly, we previously reported an SR of 97% surgical specimens and 74% in small biopsies tested using a panel of 324 cancer genes [12]. Surprisingly, RNA-seq data appear to be different, with an SR of 48.7% in surgical specimens and 60.3% in small biopsies. Therefore, better results were obtained with small biopsies than with surgical or cell block specimens (*p* = 0.002). This difference is probably due to warm and cold ischemia, which compromise RNA molecules during and after surgical intervention, respectively [9,13,14]. Among biopsies, US-guided and EBUS/EUS biopsies performed slightly better than the thoracoscopic and CT-guided biopsies, achieving an SR of 64.7% and 62.5% vs. 55.5% and 50%, respectively. We speculated that the advantage of US-guided and EBUS/EUS techniques relies on the capability to sample the periphery of the lesion, avoiding areas of necrosis and fibrosis that can affect the quality and quantity of RNA. Although no differences were found in terms of the RNA fragmentation index (*p* = 0.967), significant differences were found in terms of the RNA concentration between biopsy procedures (*p* = 0.017). Considering the surgical specimens, despite the overall SR being 48.7%, we noted that the samples from lobectomies had an SR of 29.4% and the samples from wedge resections had an SR of 66.6%. The differences in SRs could be due mainly to the timing of the interventions. Wedge resections are fast procedures compared with lobectomies and are characterized by shorter warm ischemia times, which was supported by the significant differences in the RNA fragmentation index between the lobectomies and wedge resections (*p* = 0.028). A warm ischemia time refers to the duration for which an organ remains at body temperature after its blood supply has been stopped, and it exclusively depends on variables related to surgical intervention, whereas cold ischemia time is defined as the period of removal of an organ (or tissue) until further preservation of the specimen, such as chemical fixation or snap freezing, and mostly depends on logistics [15]. Our findings suggest that a warm ischemia time could be a crucial pre-analytical variable in RNA-seq. No data were obtained from RNA-seq using samples from cell blocks, and the cohort is too limited for further consideration.

Recent findings support the idea that storage time can compromise molecular evaluations [14,15,16,17,18,19,20]. In our previous study, we demonstrated that, in samples tested using DNA-seq, the longer the sample had been stored, the lower the SR, with the best results obtained within the first 6 months [12]. In the present study, we focused on the effects of storage time on RNA and demonstrated that RNA fragmentation inversely correlates with storage time (*p* = 0.037; r = −0.206). Moreover, we noted a trend in which the SR decreases with time. Thus, the role of storage time on RNA molecules should be investigated further to define the optimal time frame for molecular testing.

As tissues that are both under-fixation and over-fixation compromise the results of molecular analysis, a total fixation time of no less than 6 h and no greater than 36 h is recommended for most tissues [9,21,22]. In line with this evidence, we found a negative correlation between the RNA fragmentation index and fixation days (*p* < 0.001; r = −0.333), indicating that over-fixed tissue specimens are suboptimal samples for RNA-seq.

With bone biopsies, tissues are commonly decalcified using acids or calcium-chelating agents before processing. Strong acid decalcification (i.e., HCl, HNO_3_) before or during the fixation process results in the hydrolysis of DNA and RNA and is contraindicated for the molecular analyses of nucleic acids; in these cases, the use of ethylenediaminetetraacetic acid (EDTA) is preferred [23]. In our series, samples obtained from bone metastasis and decalcified using EDTA were suitable for NGS analysis (SR 50%), which supports the finding that EDTA allows for the detection of gene mutations, amplifications, and even fusion transcripts [23].

The main limitation of the present study was the inability to evaluate the impact of the intrinsic variability of our methods. We performed all of the analyses (RNA-seq and RNA concentration/fragmentation evaluation) by adopting a single platform. Moreover, as our cohort was imbalanced with biopsies and surgical specimens, more studies are needed to assess the role of pre-analytics on RNA in cell blocks.

To the best of our knowledge, the present study shows for the first time the role of sample type (i.e., biopsy or surgical specimen, wedge resection or lobectomy) in the SR of RNA-seq. Moreover, our data suggest a shorter time frame for RNA fragmentation in FFPE tissues rather than DNA. This evidence underlines the need to define personalized approaches in NGS that take into account pre-analytical variables related to the specific molecular testing technique that is adopted (DNA- or RNA-based).

## 5. Conclusions

Despite RNA-seq being considered the gold standard for fusion detection in NSCLC, its reliability in clinical practice is strictly dependent on RNA integrity. Few data are available on the effects of pre-analytical variables on RNA-seq. In the present study, we evaluated the variables that affected RNA integrity for eligibility in RNA-based NGS. These findings are pivotal in the choice of the more suitable sample for RNA-seq when two or more specimens are available from the same patient.

According to our findings, NSCLC samples with a high RNA concentration and fragmentation index, with a low fixation and storage time, obtained from small biopsies, wedge resections or metastasectomy, should be preferred to be tested using RNA-seq.

Knowing more about pre-analytical variables that affect molecular testing will allow the improved application of personalized medicine, both in real-life clinical practice and clinical studies.

## Figures and Tables

**Table 1:** Baseline characteristics of 140 tissue samples.

Site			N
T			102
N			14
M			24
**Sample type**			
Biopsy			69
Surgical specimen	Lobectomy	34	63
	Wedge resection	18	
	Metastasectomy	11	
Cell block			8
**Diagnosis**			
Adk			124
Non-Adk, Non-SqCC			14
SqCC			2

Adk: adenocarcinoma. SqCC: squamous cell carcinoma.

**Table 2:** Success rate (SR) of RNA-seq tested samples based on pre-analytical variables.

Site			SR
T			39/73 (53.4%)
N			4/11 (36.4%)
M	Bone	2/4 (50%)	11/18 (61.1%)
	Extraskeletal	9/14 (64%)	
**Sample type**			
Cell block			0/5 (0%)
Biopsy	US-guided	11/17 (64.7%)	35/58 (60.3%)
	EBUS/EUS	10/16 (62.5%)	
	Thoracoscopic	5/9 (55.5%)	
	CT-guided	6/12 (50%)	
Surgical specimen	Lobectomy	5/17 (29.4%)	19/39 (48.7%)
	Wedge resection	10/15 (66.6%)	
	Metastasectomy	4/7 (57.1%)	
**Fragmentation** **index**			
≥0.14			42/55 (76.4%)
<0.14			12/47 (25.5%)
**Storage time (days)**			
≤30			22/33 (66.6%)
>30			32/69 (46.4%)

## Data Availability

Patient data were registered using a univocal ID code, which is potentially traceable; therefore, data are unavailable due to privacy.

## Supplementary Materials

The following supporting information can be downloaded at: https://www.mdpi.com/article/10.3390/jpm14080851/s1; Table S1. RNA concentration isolated from each NSCLC sample.
