# Supplementary material for: RNA-Seq Analysis in Non-Small Cell Lung Cancer: What Is the Best Sample from Clinical Practice?
Source: J Pers Med. 2024 Aug 11;14(8):851. doi: 10.3390/jpm14080851 (PMC11355753; doi:10.3390/jpm14080851)
Supplement: Supplementary file 1 [file jpm-14-00851-s001.zip › jpm-3103637-supplementary.pdf]

Table S1. RNA concentration isolated from each NSCLC sample

[] RNA [ng/ul]			
0,82	14,86	11,18	152
3,26	20,1	47	29,4
0,00	0,72	16,9	27
93	71,13	22	35,8
11,2	0,00	0,22	334,6
6,46	0,07	0,34	
161,8	2,84	5,63	
6,40	10,44	7,34	
6,46	25	6,9	
3,74	4,24	14,71	
4,91	7,41	9,75	
18,06	8,50	35	
0,63	31,25	0,67	
13,73	0,33	0,03	
2,3	38,7	38,39	
0,32	3,7	50,5	
8,56	66	1,24	
5,6	2,31	12,8	
33	13,00	0,10	
0,086	33,7	27,25	
76,7	41	54	
22	16,87	0,24	
0,00	57,9	0,36	
10,44	1,5	0,42	
10	0,04	27	
151	0,21	9,39	
0,89	0,32	12,9	
18	1,34	54,47	
0,15	4,00	5,63	
0,00	2,01	11	
0,03	7,41	3,74	
0,15	0,02	0,77	
10,44	13,73	0,24	
20,29	0,72	8	
12,8	0,008	12,57	
20,72	0,36	0,54	
120,00	2,83	168,00	
41,1	54	141	
11,93	0,01	22	
54	0,44	81,6	
15,7	0,0003	1,16	
0,03	0,0118	2,74	
2,8	0,0191	4,2	
18,2	22	4,90	
0,28	2,8	13,70	
